# Platelet-derived growth factor (PDGF)-BB-mediated induction of monocyte chemoattractant protein 1 in human astrocytes: implications for HIV-associated neuroinflammation

**DOI:** 10.1186/1742-2094-9-262

**Published:** 2012-12-01

**Authors:** Crystal Bethel-Brown, Honghong Yao, Guoku Hu, Shilpa Buch

**Affiliations:** 1 Department of Pharmacology and Experimental Neuroscience, University of Nebraska Medical Center, Omaha, NE 68198, USA; 2 Department of Medical Microbiology and Immunology, Creighton Medical Center, Omaha, NE 68178, USA; 3 Department of Pharmacology and Experimental Neuroscience, 985880 Nebraska Medical Center (DRC 8011), University of Nebraska Medical Center, Omaha, NE 68198-5880, USA

## Abstract

Chemokine (C-C motif) ligand 2, also known as monocyte chemoattractant protein 1 (MCP-1) is an important factor for the pathogenesis of HIV-associated neurocognitive disorders (HAND). The mechanisms of MCP-1-mediated neuropathogenesis, in part, revolve around its neuroinflammatory role and the recruitment of monocytes into the central nervous system (CNS) via the disrupted blood-brain barrier (BBB). We have previously demonstrated that HIV-1/HIV-1 Tat upregulate platelet-derived growth factor (PDGF)-BB, a known cerebrovascular permeant; subsequently, the present study was aimed at exploring the regulation of MCP-1 by PDGF-BB in astrocytes with implications in HAND. Specifically, the data herein demonstrate that exposure of human astrocytes to HIV-1 LAI elevated PDGF-B and MCP-1 levels. Furthermore, treating astrocytes with the human recombinant PDGF-BB protein significantly increased the production and release of MCP-1 at both the RNA and protein levels. MCP-1 induction was regulated by activation of extracellular-signal-regulated kinase (ERK)1/2, c-Jun N-terminal kinase (JNK) and p38 mitogen-activated protein (MAP) kinases and phosphatidylinositol 3-kinase (PI3K)/Akt pathways and the downstream transcription factor, nuclear factor κB (NFκB). Chromatin immunoprecipitation (ChIP) assays demonstrated increased binding of NFκB to the human MCP-1 promoter following PDGF-BB exposure. Conditioned media from PDGF-BB-treated astrocytes increased monocyte transmigration through human brain microvascular endothelial cells (HBMECs), an effect that was blocked by STI-571, a tyrosine kinase inhibitor (PDGF receptor (PDGF-R) blocker). PDGF-BB-mediated release of MCP-1 was critical for increased permeability in an *in vitro* BBB model as evidenced by blocking antibody assays. Since MCP-1 is linked to disease severity, understanding its modulation by PDGF-BB could aid in understanding the proinflammatory responses in HAND. These results suggest that astrocyte activation by PDGF-BB exaggerates monocyte recruitment into the brain via MCP-1 and underscores the critical role astrocytes play in HAND.

## Background

HIV-associated neurocognitive disorders (HAND) remain a common complication of HIV infection affecting up to 60% of infected individuals despite the use of antiretroviral therapy (ART) [1]. With the advancement of ART the prevalence of HAND has actually increased, partly due to both increased survival rates of HIV-infected individuals and to the reduced ability of most of these drugs to cross the blood–brain barrier (BBB). Among the factors involved in the pathogenesis of HAND, influx of HIV-infected monocytes in response to the chemokine monocyte chemoattractant protein 1 (MCP-1) via a breached endothelial barrier, plays a critical role in disease pathogenesis. MCP-1 plays a vital role in the recruitment of monocytes into the brain contributing to neuroinflammation and BBB disruption [2,3]. This chemokine has been extensively studied and is expressed by a number of cell types including astrocytes, microglia and neurons [4,5]. Elevated expression of MCP-1 has been demonstrated in various diseases including multiple sclerosis, amyloid lateral sclerosis, lupus nephritis, peripheral neuropathy and Alzheimer’s disease [6-13]. While increased expression of MCP-1 has been shown to correlate with HIV-associated central nervous system (CNS) complications, regulation of this chemokine in the context of HIV disease remains less clear. Understanding the molecular mechanisms modulating MCP-1 may thus provide insights into development of therapeutic targets for many neurodegenerative diseases including HAND.

Platelet-derived growth factor (PDGF) is a well known and potent inducer of MCP-1. The PDGF family of proteins is very closely related to the vascular endothelial growth factor (VEGF) family and is highly conserved throughout the animal kingdom [14]. These proteins are usually expressed as dimers: PDGF-A and PDGF-B can form homodimers or heterodimers, and PDGF-C and PDGF-D form homodimers. For the sake of clarity, in this study, PDGF-B refers to the RNA expression, whereas PDGF-BB refers to the protein expression of these genes. Many studies on PDGF have focused primarily on its mitogenic effects [15-17], however, divergent effects of PDGF are rapidly emerging. For example, recent studies by Lawrence *et al.* have demonstrated PDGF to be a cerebrovascular permeant that can disrupt BBB integrity during ischemic stroke conditions [18]. Along similar lines, it has been shown that PDGF-BB can disrupt BBB via the modulation of molecules important in maintaining tight junctions such as ZO-1 and adhesion molecules [19].

Since astrocytes are a major source of MCP-1 in the brain and PDGF-BB has been shown to be an inducer of MCP-1, the purpose of this study was to explore the modulation of MCP-1 by PDGF-BB released from HIV-treated astrocytes. We hypothesize that PDGF-BB induced by HIV-1/HIV-1 Tat can result in astrocytic activation and release of MCP-1 and BBB disruption. The data demonstrate that the exposure of human astrocytes to HIV-1 LAI resulted in the induction of PDGF at both the mRNA and protein levels. To explicate the mechanism/s involved in PDGF-BB/MCP-1 interaction, human astrocytes were then treated with PDGF-BB and monitored for expression of MCP-1. Utilizing pharmacological and genetic approaches we demonstrate the involvement of extracellular-signal-regulated kinase (ERK)1/2, c-Jun N-terminal kinase (JNK) and p38 mitogen-activated protein kinases (MAPKs), Phosphatidylinositol 3-kinase (PI3K)/Akt pathways and the transcription factor nuclear factor κB (NFκB) in PDGF-BB-mediated induction of MCP-1 in astrocytes. Because both PDGF-BB and MCP-1 are known to affect the BBB and since astrocytic end-feet processes are in close contact with the endothelia, we also addressed the functional implications this may have on the BBB. Using pharmacological and neutralizing antibody approaches, we reveal that both PDGF-BB and MCP-1 play critical roles in reducing the integrity of the BBB. These data highlight the role of PDGF-BB in astrocytic release of MCP-1, which in turn, is critical for recruitment of monocytes across the BBB. Taken together, these studies underscore the role of PDGF signaling as a potential therapeutic target of HAND.

## Materials and methods

### Materials

Recombinant human PDGF-BB was purchased from R&D Systems (Minneapolis, MN, USA). All experiments involving the treatment of cells with exogenous PDGF-BB were conducted under serum-free conditions because serum induces PDGF. STI-571, an inhibitor of tyrosine kinase receptors, was obtained from Novartis (Basel, Switzerland). The specific phosphatidinylinositol-3’ kinase (PI3K) inhibitor LY294002, mitogen-activated protein (MAP) kinase kinase (MEK) inhibitor U0126 and p38 mitogen activated kinase (p38) inhibitor SB203558 were purchased from Promega (Madison, WI, USA). The JNK inhibitor SP600125 was purchased from Assay Designs (Ann Arbor, MI, USA). MCP-1 neutralizing antibody was obtained from eBioscience (San Diego, CA, USA). The C-C chemokine receptor type 2 (CCR2) antagonist, RS 102895, was purchased from Sigma (St Louis, MO, USA). The chromatin immunoprecipitation (ChIP) assay kit was obtained from Upstate, (Billerica, MA, USA).

### Cell culture and cell lines

The human astrocytic cell line A172 (no. CRL-1620; American Type Culture Collection (ATCC)) were cultured as described previously [20] and maintained in Dulbecco's modified Eagle’s medium (DMEM) high glucose medium containing 10% heated-inactivated fetal bovine serum, 2 mM glutamine, penicillin (100 units/ml), streptomycin (100 μg/ml), essential amino acids and vitamins. In this study, A172 cells were used within 30 passages. Human primary astrocytes were obtained from ScienCell Research Laboratories (Carlsbad, CA, USA) and were cultured in DMEM/F12 medium (Invitrogen Life Technologies, Carlsbad, CA, USA) containing 10% heat-inactivated fetal bovine serum (FBS), 2 mM glutamine, sodium bicarbonate, gentamicin, non-essential amino acids and vitamins. Primary human brain microvascular endothelial cells (HBMECs) obtained from Dr Monique Stins (The Johns Hopkins University, Baltimore, MD) were cultured in RPMI 1640 medium containing 10% heat-inactivated fetal bovine serum, 10% Nu-Serum, 2 mM glutamine, 1 mM pyruvate, penicillin (100 U/ml), streptomycin (100 μg/ml), essential amino acids, and vitamins according to our previous publication [21].

### Cell treatment

Human astrocytes were serum starved overnight prior to treatment. The HIV-1 LAI used in this study was propagated in stimulated peripheral blood mononuclear cells (PBMCs) [22] (supplied by Dr Howard Gendelman, University of Nebraska Medical Center, Omaha, NE, USA). The rationale for using a C-X-C chemokine receptor type 4 (CXCR-4)-tropic virus is based upon previous studies demonstrating the susceptibility of astrocytes to CXCR4-tropic viruses [23,24]. In the pharmacological inhibitor studies, the cells were pretreated with inhibitors specific for MEK (U0126, 1 μM), JNK (SP600125, 10 μM), P38 (SB20358, 1 μM) and PI3K (LY294002, 1 μM) for 1 h prior to PDGF-BB exposure. The inhibitors were not removed from the astrocytes during PDGF-BB treatment and concentrations utilized in this study were based upon our previous studies [25].

### MCP-1 protein analysis by enzyme-linked immunosorbent assay (ELISA)

MCP-1 levels were examined using an MCP-1 ELISA kit purchased from R&D Systems. Samples were analyzed for MCP-1 protein according to the manufacturer’s instructions in triplicates determined in at least three independent experiments.

### Reverse transcription and real-time RTPCR (RT-PCR)

Total RNA was extracted using Trizol reagent according to the manufacturer’s protocol (Invitrogen). RNA (1 μg) was used for cDNA production according to manufacturer’s instructions (Thermo Scientific, Waltham, MA, USA). The sequences of primers used for human MCP-1 (GenBank accession number NM 002982) were as follows: sense: CAGCAGGTGTCCCAAAGAAGCTGT antisense: CCATTCCTTATTGGGGTCAGCACAGA. The sequences of primers used for glyceraldehyde-3-phosphate dehydrogenase (GAPDH) (GenBank accession number NM 002046) were as follows: sense: TGCACCACCAACTGCTTAGC; antisense: GGCATGGACTGTGGTCATGAG. The primers for CCR2 (GenBank accession number NM 001123396) were as follows: first round sense: TCTGGAGACCTCAACCAAATG; antisense: GGAAATGCGTCCTTGTTCAA. The primers used in the second round were as follows: sense: CCCTGTATCTCCGCCTTCACT; antisense: TTCAGCTTGTGGCTTGTCTCA. Quantitative analyses of mRNA were conducted using an ABI 7500 Fast Real-Time PCR system (Applied Biosystems, Foster City, CA, USA). The primers for PDGF-B, MCP-1 and 18S were purchased from SA Biosciences (Frederick, MD, USA). Data were normalized using Ct values for GAPDH or 18S in each sample. To calculate relative amounts mRNA, the average Ct values were subtracted from GAPDH or 18S values for each target gene to provide changes in Ct value. Fold change in expression was calculated as log_2_ relative units.

### Flow cytometry

Untreated human primary astrocytes and A172 human astrocytes were collected in cold phosphate-buffered saline (PBS) and ethylenediaminetetra-acetic acid (EDTA) (5 mM) followed by incubation with anti-PDGF receptor α (PDGF-Rα) (CD140α; 1:5) and anti-PDGF-Rβ (CD140β; 1:5) BD LSR II (BD Biosciences, San Jose, CA, USA) was used for fluorescence acquisition, and data were analyzed with FACS Diva software (BD Biosciences).

### Western blotting

PDGF-BB treated astrocytes were lysed using the Mammalian Cell Lysis kit (Sigma) and the NE-PER Nuclear and Cytoplasmic Extraction kit (Pierce, Rockford, IL, USA) as per manufacturer’s instructions. Cell lysates were subjected to separation by 12% sodium dodecyl sulfate polyacrylamide gel electrophoresis (SDS-PAGE) (30 μg protein per well) and transferred to polyvinylidene difluoride (PVDF) membranes. The blots were blocked with 5% non-fat dry milk in PBS. Western blots were then probed with antibodies recognizing phosphorylated forms of ERK1/2, JNK, p38 and Akt and total forms of ERK1/2, JNK, and Akt (Cell Signaling, Danvers, MA, USA; 1:500); NFκB-p65 and phosphorylated inhibitor of κB (pIκB) Cell Signaling, (1:1,000); Histone (1:1,000) and β-actin antibodies (Santa Cruz Biotechnologies, Santa Cruz, CA, USA; 1:5,000). Signals were detected by chemiluminescence (Pierce).

### Transduction with adenoviral constructs

A172 cells were infected with adenoviral constructs containing either wild-type (WT) or dominant-negative (DN) forms of Akt (a kind gift from Dr K Walsh, Tufts University School of Medicine, Boston, MA, USA). In addition, cells were also transduced with recombinant adenoviral vectors expressing full-length p65/RelA or p65/RelA mutant (RelA 1–300) (a kind gift from Dr S Maggiwar, University of Rochester Medical Center, Rochester, NY, USA) used at a multiplicity of infection (MOI) of 50 as previously described [26]. Astrocytes infected for 24 h with adenoviral constructs were subsequently treated with PDGF-BB, followed by assessment by western blotting or ELISA.

### Short interfering RNA transfection of astrocytes

**Figure 1:**
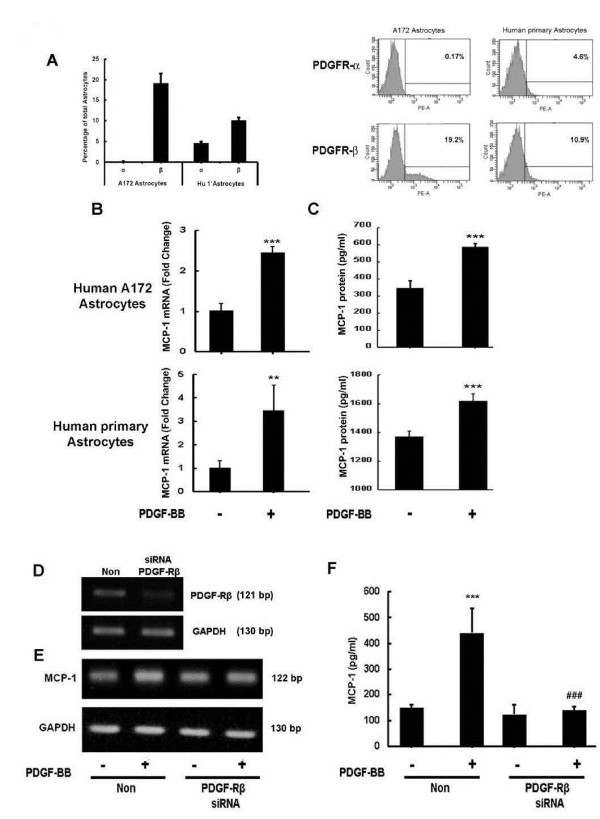
**Engagement of platelet-derived growth factor receptor (PDGF-R) is critical for PDGF-BB induced monocyte chemoattractant protein 1 (MCP-1) expression in astrocytes. **(**A**) Human primary as well as A172 primary astrocytes express both PDGF-α and PDGF-β receptors. Untreated human primary and A172 astrocytes were subjected to flow cytometric analyses using antibodies against human PDGF-αR (CD140α) and PDGF-βR (CD140β). (**B**,**C**) PDGF-BB-mediated induction of MCP-1 mRNA and protein expression in human primary and A172 astrocytes. (B) Human primary and A172 astrocytes were exposed to PDGF-BB for 6 h. Total RNA isolated from human A172 astrocytes was subjected to real-time reverse-transcriptase polymerase chain reaction (RT-PCR) analysis using primers for human MCP-1. (**C**) Supernatant fluid from astrocytes treated with PDGF-BB for 24 h were assessed for release of chemokine MCP-1 using the human MCP-1 enzyme-linked immunosorbent assay (ELISA) kit. (**D**) Total RNA from A172 cells transfected with either nonsense (Non) or PDGF-Rβ short interfering (si)RNAs was subjected to RT-PCR analysis using primers specific for PDGF-Rβ. (**E**) siRNA, but not Non siRNA inhibited PDGF-BB-mediated induction of MCP-1 RNA. (**F**) Supernatants from A172 cells transfected with either Non or siRNAs were subjected to MCP-1 ELISA assay. PDGF-Rβ siRNA, but not Non siRNA inhibited PDGF-BB-mediated induction of MCP-1 protein. **P <0.01, ***P <0.001 versus control group.

Short interfering RNA (siRNA) targeted against PDGF-Rβ was obtained from Dharmacon (Boulder, CO, USA). Human A172 cells were plated in 24-well plates at a density of 4 × 10^4^ cells per well 1 day prior to transfection. Cell culture medium was replaced with 250 ml pre-warmed OPTI-MEM I culture medium. Lipofectamine-2000 reagent (Invitrogen) was then combined with serum-free medium for 5 minutes at room temperature. The PDGF-Rβ siRNA was then added into the mixture described above to a final concentration of 5 μM. Then, siRNA and the reagent mixture were incubated for 20 minutes at room temperature, after which, the combined mixture was added to the cells. The cell culture plate was shaken gently for 5 s and incubated for 24 h at 37°C. Knockdown efficiency (Figure 1D) of the transfected astrocytes were (54%) as determined by RT-PCR.

### Transfection with plasmid constructs

DN and WT constructs of MEK were provided by Dr Young Han Lee (Konkuk University, Korea). A172 cells were transfected with plasmid constructs containing either WT or DN forms of MEK as described above. The transfection efficiency was 42% as determined by immunostaining (data not shown).

### ChIP assay

The ChIP assay was performed according to the manufacturer’s instructions (Upstate, Billerica, MA, USA) with slight modifications. After treatment of the cells, 18.5% fresh formaldehyde was added directly into the medium at a final concentration of 1% formaldehyde and incubated for 10 minutes at room temperature, followed by quenching with 125 mM glycine. The cells were then scraped using 2 ml pre-chilled PBS containing 1 × protease inhibitor mixture. The cell pellet was harvested by spinning at 800 *g* at 4°C, and lysis buffer was added (provided in the kit) to harvest nuclei. DNA was then sheared by sonication. A total of 50 μl of the sheared crosslinked chromatin was then mixed with 20 ml protein A magnetic beads and 5 mg of immunopreciptating Abs against NFκB p65, acetyl histone H3 (as a positive control), and normal rabbit IgG (as a negative control) diluted in 450 ml dilution buffer overnight at 4°C. The magnetic beads binding Ab-chromatin complex was then washed with 0.5 ml each of a series of cold wash buffers in the order of low salt buffer, high salt buffer, LiCl buffer, and Tris-EDTA buffer. The crosslinking of protein-DNA complexes were reversed to free DNA by incubation at 62°C for 2 h and purified using DNA purification spin columns following the manufacturer’s instructions. Finally, the purified DNA was amplified (35 cycles) via PCR to identify the promoter region containing NFκB binding site ‘GGGCCTTTCC’. The sequence of the primers used to identify the MCP-1 promoter bound to NFκB were as follows: sense: GCATCAGAGCATTGACCCTCA; antisense: AGGTCAGTGCTGGCGTGAGA.

### Monocyte isolation and transmigration

Primary HBMECs seeded on 6.5 mm polyester transwell inserts (5.0 μm pore size) were grown to confluence. Following confluency, conditioned media from primary astrocytes treated with PDGF-BB was added to the bottom chamber of the transwell and allowed to incubate at 37°C in a humid atmosphere of 5% CO_2_ for 24 h. MCP-1 neutralizing antibody was added to PDGF-BB treated conditioned media prior to HBMEC exposure for 24 h. Monocytes were obtained from HIV-1, HIV-2 and hepatitis B seronegative donor leukopacks, and separated by countercurrent centrifugal elutriation as previously described [27]. Monocytes were washed with PBS and fluorescently labeled with 10 μM Cell tracker green (Invitrogen) for 10 minutes at room temperature. Labeled cells (2 × 10^5^ cells) were added to the upper compartments of transwell inserts and allowed to transmigrate at 37°C in a humid atmosphere of 5% CO_2_ for 24 h. Transmigrated monocytes were quantified using florescent plate reader (435 nm/528 nm excitation/emission).

### Statistical analysis

Statistical analysis was performed using one-way analysis of variance (ANOVA) with a *post hoc* Student’s t test. Results were judged statistically significant if *P* <0.05 by analysis of variance.

## Results

### HIV-1-mediated upregulation of PDGF-B and MCP-1 in astrocytes

**Figure 2:**
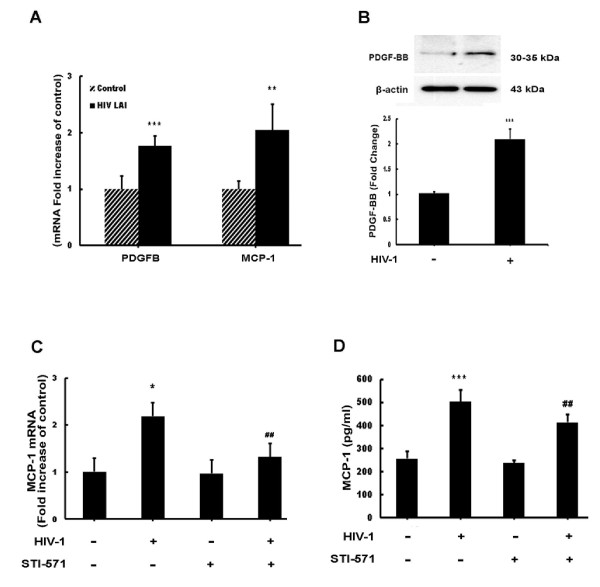
**HIV-1 induces platelet-derived growth factor (PDGF)-B and monocyte chemoattractant protein 1 (MCP-1) mRNA and protein expression in astrocytes. **(**A**) HIV-1 LAI induced PDGF-B chain and MCP-1 mRNA expression in human A172 astrocytes. Total RNA isolated from human A172 astrocytes was subjected to real-time reverse-transcriptase polymerase chain reaction (RT-PCR) analysis using primers specific for human PDGF-B, MCP-1 and 18S. (**B**) HIV-1 LAI induces PDGF-BB protein expression in human A172 astrocytes. Protein lysates isolated from astrocytes exposed to HIV-1 were subjected to western blot analysis and analyzed for expression of PDGF-BB protein. (**C**) HIV-1 LAI induces MCP-1 mRNA levels. Total RNA isolated from human A172 astrocytes was subjected to RT-PCR analysis using primers specific for human MCP-1 and 18S. (**D**) HIV-1 LAI induces MCP-1 protein expression in human A172 astrocytes. Supernatants from astrocytes exposed to HIV-1 for 24 h were subjected to MCP-1 enzyme-linked immunosorbent assay (ELISA) analysis. All the data are presented as mean ± SD of three individual experiments. *P <0.05, **P <0.01, ***P <0.001 versus control group; ##*P *<0.01 versus HIV-1-treated group.

Since astrocytes in the CNS are exposed to HIV-1, we first sought to examine the modulation of PDGF-B and MCP-1 by HIV-1. Purified HIV-1 LAI virus obtained by high-speed ultracentrifugation and resuspended in astrocyte serum-free media was used for these experiments. Serum-starved astrocytes were exposed to purified virus at a MOI of 0.1 for 6 h followed by assessment of RNA levels by real-time RT-PCR. The MOI of HIV-1 LAI used was based upon our previous study [25]. As shown in Figure 2A, HIV-1 LAI significantly upregulated both PDGF-B (1.7-fold) and MCP-1 (twofold) mRNA levels. To confirm whether increased mRNA levels of PDGF-B translated into increased protein, a western blot analysis was performed on lysates of astrocytes exposed to HIV-1 LAI for 24 h. As shown in Figure 2B exposure to HIV-1 LAI also induced upregulation of PDGF-BB protein. Likewise, supernatants from A172 cells treated with HIV LAI were analyzed for MCP-1 levels via ELISA. As shown in Figure 2D, HIV-1 LAI exposure also resulted in increased MCP-1 levels. To determine whether HIV-mediated induction of MCP-1 could, in part, be explained due to increased PDGF-BB levels, astrocytes were treated with the PDGF receptor blocker STI-571 for 1 h prior to HIV-1 exposure and assessed for MCP-1 expression. Blocking the PDGF-R significantly reduced HIV-mediated induction of MCP-1 RNA (Figure 2C) and protein (Figure 2D).

### PDGF-BB-mediated upregulation of MCP-1 in astrocytes

**Figure 3:**
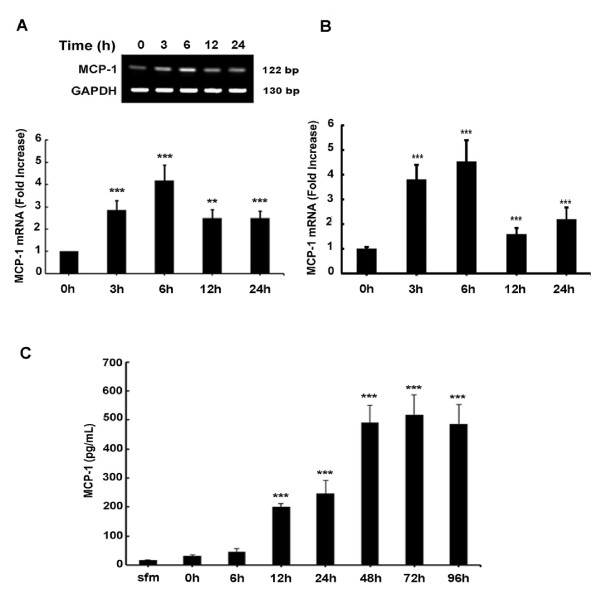
**Platelet-derived growth factor (PDGF)-BB induces monocyte chemoattractant protein 1 (MCP-1) mRNA and protein expression in human astrocytes. **(**A**,**B**) Time-dependence of PDGF-BB-mediated induction of MCP-1 mRNA expression in human A172 astrocytes. Total RNA isolated from human A172 astrocytes was subjected to reverse-transcriptase polymerase chain reaction (RT-PCR) and real-time RT-PCR analysis using primers for human MCP-1 and glyceraldehyde-3-phosphate dehydrogenase (GAPDH) and 18S, respectively. PDGF-BB-mediated induction of MCP-1 mRNA expression peaked at 6 h and decline thereafter. (**C**) Time-dependence of PDGF-BB-mediated induction of MCP-1 protein expression in human A172 astrocytes. Supernatant fluid from human A172 cells treated with PDGF-BB for various time points were assessed for release of chemokine MCP-1 using the human MCP-1 enzyme-linked immunosorbent assay (ELISA) kit. PDGF-BB treatment resulted in a time-dependent induction of MCP-1 expression. All the data are presented as mean ± SD of three individual experiments. **P <0.01, ***P <0.001 versus control group; sfm = serum-free media.

All experiments involving the treatment of cells with exogenous PDGF-BB protein were conducted under serum-free conditions since PDGF promoter is known to have serum response elements [28,29]. To investigate the role of PDGF on MCP-1 expression, A172 cells were treated with recombinant PDGF-BB (20 ng/ml) for the indicated times and mRNA levels were assessed by RT-PCR and real-time RT-PCR. The concentration of PDGF-BB used was based on previous studies [25,30]. Following exposure of A172 astrocytes to PDGF-BB, there was a time-dependent upregulation of MCP-1 mRNA compared with untreated cells (Figure 3A) with a peak induction at 6 h post treatment (4.5-fold) and a subsequent downregulation at 12 h. Similar results were obtained via real-time PCR (Figure 3B).

To determine whether the increased MCP-1 mRNA expression correlated with increased protein release in astrocytes, we next examined the MCP-1 protein levels. Cells were serum-starved as described and treated with PDGF-BB for 0 to 96 h. Supernatant fluids were collected and MCP-1 protein levels were assessed by ELISA. As shown in Figure 3C, PDGF-BB upregulated MCP-1 protein levels in a time-dependent manner with a continual increase that plateaued at 48 h post treatment. Cumulatively, these data clearly demonstrate that PDGF-BB treatment mediated the induction of MCP-1 RNA and protein in astrocytes.

### Engagement of PDGF-Rβ is critical for PDGF-induced MCP-1 expression in astrocytes

Because PDGF-BB mediates its effects via binding to its cognate receptors PDGF-Rα and PDGF-Rβ, our next step was to examine the role of receptor binding in PDGF-BB-induced MCP-1 release by astrocytes. However, before proceeding with this, it was important to first examine whether astrocytes indeed expressed PDGF-Rα and PDGF-Rβ. Flow cytometric analysis of A172 and human primary astrocytes revealed a stark difference between the two types of cells. As shown in Figure 1A, of the A172 cells examined, endogenous levels of PDGF-Rα and PDGF-Rβ were 0.17% and 19.2%, respectively. Human primary astrocytes expressed higher levels of PDGF-Rα (4.6%) and lower PDGF-Rβ (10.9%) levels compared to A172 astrocytes. Despite the difference in relative distribution of the PDGF receptors, PDGF-Rβ was the major receptor form expressed in both types of astrocytes utilized in this study. Similar results were observed by RT-PCR (data not shown).

Having determined the relative expressions of PDGF-Rs in both human primary and A172 astrocytes, we next sought to confirm the effect of PDGF-BB on MCP-1 release in both cells types. Astrocytes were exposed to PDGF-BB for 6 h and 24 h for mRNA and protein assessment, respectively. As shown in Figure 1B, PDGF-BB upregulated MCP-1 mRNA levels in both human A172 as well as human primary astrocytes. MCP-1 protein ELISA analysis also corroborated this upregulation, as shown in Figure 1C.

To determine the role that the PDGF-Rs played in PDGF-BB-mediated MCP-1 release from astrocytes, human A172 astrocytes were transfected with PDGF-Rβ siRNA followed by treatment of PDGF-BB and MCP-1 levels were assessed 24 h later. As shown in Figure 1D, transfection of human A172 cells with PDGF-Rβ siRNA resulted in efficient knockdown of PDGF-Rβ as demonstrated by RT-PCR. Cells were transfected with either PDGF-Rβ siRNA or non-specific (Non) siRNA control followed by treatment with PDGF-BB for 24 h. PDGF-BB-mediated induction of MCP-1 mRNA was attenuated in cells transfected with PDGF-Rβ siRNA but not in the Non siRNA controls cells (Figure 1E). These results were further confirmed by examining PDGF-BB-mediated induction of MCP-1 protein levels. As shown in Figure 1F and as expected, PDGF-BB treatment resulted in enhanced release of MCP-1 protein in Non siRNA transfected cells but not in cells transfected with PDGF-Rβ siRNA. Taken together, these findings confirmed the involvement of PDGF-Rs in PDGF-BB-mediated induction of MCP-1 in astrocytes.

### PDGF-BB-mediated induction of MCP-1 involves MAPK and PI3K/Akt cell signaling pathways

**Figure 4:**
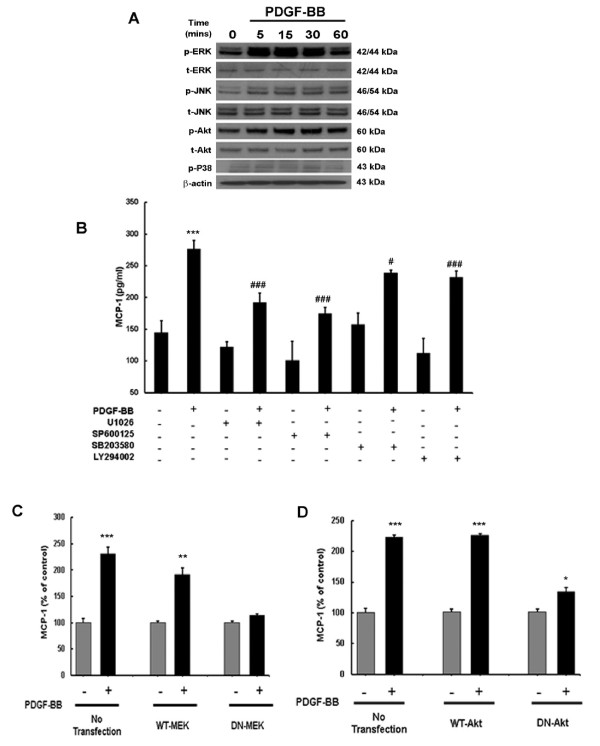
**Platelet-derived growth factor (PDGF)-BB-mediated induction of monocyte chemoattractant protein 1 (MCP-1) expression involves mitogen-activated protein kinases (MAPKs) and phosphatidylinositol 3-kinase (PI3K)/Akt cell signaling pathways. **(**A**) Western blot analysis of time-dependent activation of extracellular-signal-regulated kinase (ERK), c-Jun N-terminal kinase (JNK), P38 and Akt by PDGF-BB. (**B**) Inhibition of the ERK, JNK, p38 and Akt pathways by mitogen-activated protein (MAP) kinase kinase (MEK)1/2 (U0126), JNK (SP600125), p38 (SB20358) and PI3K (LY294002) inhibitors resulted in amelioration of PDGF-BB-mediated induction of MCP-1 expression in astrocytes. (**C**) Transfection with dominant-negative (DN)-MEK but not wild-type (WT)-MEK resulted in abrogation of PDGF-BB-mediated induction of MCP-1. (**D**) Transduction with DN-Akt but not WT-Akt also resulted in abrogation of PDGF-BB-mediated induction of MCP-1. All the data are presented as mean ± SD of three individual experiments. *P <0.05, **P <0.01, ***P <0.001 versus control group; #*P *<0.05, ###*P *<0.001 versus PDGF-BB-treated group.

Having determined PDGF-BB-mediated induction of MCP-1, we next sought to elucidate the signaling pathways involved in this process. Since PDGF-BB is a mitogen and a pro-survival protein, we examined the involvement of MAPK and phosphoinositide-3-kinase/Akt pathways. Human A172 cells were exposed to PDGF-BB and phosphorylation of MAPK and PI3K/Akt signaling molecules was assessed by western blotting. Treatment of astrocytes with PDGF-BB resulted in a time-dependent increase in phosphorylation of ERK1/2, JNK, p38 and Akt, with maximal activation at 30 minutes following treatment (Figure 4A). Next, to address the functional implication of MAPK and PI3K/Akt pathways in PDGF-mediated induction of MCP-1 expression, A172 cells were pretreated with inhibitors specific for the respective signaling pathways followed by PDGF-BB treatment for 24 h and subsequently assessed for expression of MCP-1. As shown in Figure 4B, treatment of cells with the MAPK and PI3K/Akt inhibitors resulted in amelioration of PDGF-BB-mediated induction of MCP-1 protein levels.

Further validation of the involvement of the MAPK pathway in this process was confirmed by transfecting cells with either the WT or DN constructs of MEK followed by 24 h treatment with PDGF-BB. PDGF-BB-mediated induction of MCP-1 was attenuated by DN-MEK, but not by WT-MEK constructs (Figure 4C). To confirm the role of PI3K/Akt in PDGF-BB-mediated MCP-1, A172 cells were transduced with adenoviral constructs containing either WT or DN forms of Akt. As shown in Figure 4D, cells transduced with DN Akt construct failed to upregulate MCP-1 unlike the cells transduced with the WT Akt construct. Taken together, these findings confirm the involvement of both MAPK and PI3K/Akt cascades in PDGF-BB-mediated induction of MCP-1 in astrocytes.

### Involvement of PDGF-Rβ in the regulation of MAPKs and PI3K/Akt cell signaling pathways

**Figure 5:**
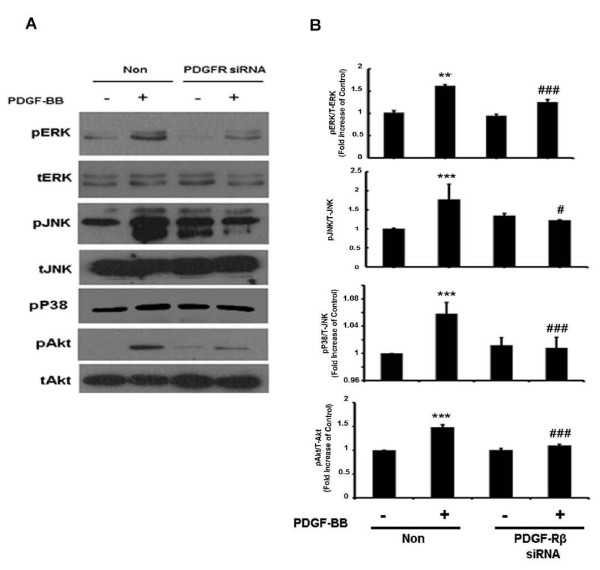
**Involvement of platelet-derived growth factor (PDGF)-Rβ in the regulation of mitogen-activated protein kinases (MAPKs) and phosphatidylinositol 3-kinase (PI3K)/Akt cell signaling pathways. **(**A**) Whole cell lysates from A172 cells transfected with either nonsense (Non) or PDGF-Rβ short interfering (si)RNAs were subjected to immunoblot analysis using antibodies specific for MAPKs and PI3K/Akt signaling. PDGF-Rβ siRNA, but not Non siRNA inhibited PDGF-BB-mediated phosphorylation of extracellular-signal-regulated kinase (ERK), c-Jun N-terminal kinase (JNK), p38 and Akt pathways. All the data are presented as mean ± SD of three individual experiments. **P <0.01, ***P <0.001 versus control group, ##*P *<0.01, ###*P *<0.001 versus PDGF-BB-treated group.

Since PDGF-BB acts through its cognate receptor, the next logical step was to link the MAPK and PI3K/Akt pathways to PDGF-R. To achieve this, siRNA was again employed. PDGF-Rβ expression was knocked down using the siRNA approach and ERK, JNK, p38, JNK and Akt phosphorylation levels were assessed. Briefly, A172 cells were transfected with PDGF-Rβ siRNA for 24 h followed by treatment with PDGF-BB for 1 h. As shown in Figure 5A,B, cells transfected with PDGF-Rβ siRNA failed to demonstrate PDGF-BB-mediated activation of ERK, JNK, p38 and Akt. These results underpin the role of PDGF-Rβ in PDGF-BB-mediated activation of MAPK and PI3K/Akt pathways.

### Involvement of NFκB in PDGF-BB-induced expression of MCP-1 in astrocytes

**Figure 6:**
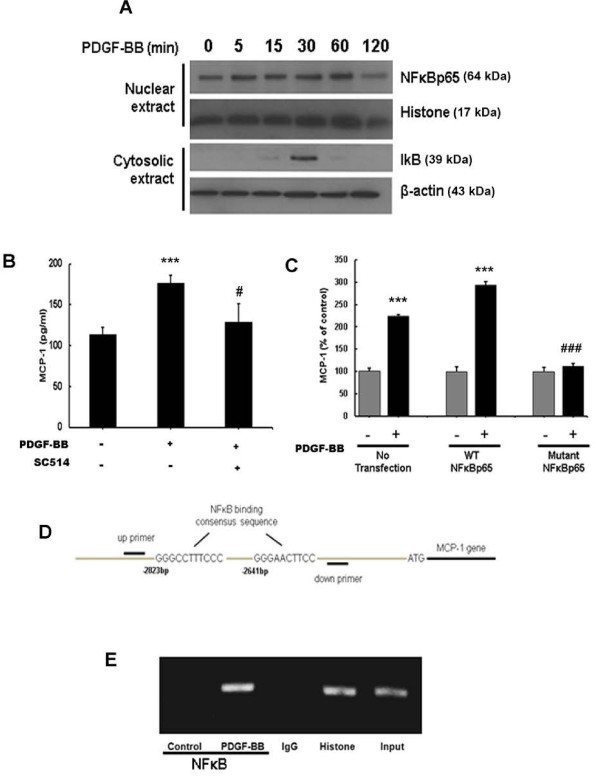
**Platelet-derived growth factor (PDGF)-BB-mediated induction of monocyte chemoattractant protein 1 (MCP-1) expression involves nuclear factor κB (NFκB) activation. **(**A**) Exposure of A172 cells to PDGF-BB resulted in a time-dependent increase in phosphorylation of the p65 subunit of NFκB in the nuclear cellular fraction. Reciprocally, PDGF-BB exposure resulted in a time-dependent increase in inhibitor of κBα (IκBα) phosphorylation in the cytosolic cellular fraction of A172 astrocytes. (**B**) Pretreatment with the IκBα inhibitor, SC514 resulted in inhibition of PDGF-BB-mediated induction of MCP-1. (**C**) Transduction with mutant-NFκB but not wild-type (WT)-NFκB resulted in abrogation of PDGF-BB-mediated induction of MCP-1. All the data are presented as mean ± SD of three individual experiments. ***P <0.001 versus control group, #*P *<0.05, ###*P *<0.001 versus PDGF-BB-treated group. (**D**) Schematic illustration of NFκB binding consensus sequence on the promoter region of MCP-1. (**E**) Chromatin immunoprecipitation (ChIP) assay demonstrating PDGF-BB-mediated binding of p65NFκB to the MCP-1 promoter.

Having determined the role of MAPK and PI3K/Akt in PDGF-BB-mediated induction of MCP-1 expression, we rationalized the involvement of NFκB in this process since this transcription factor is downstream of the aforementioned signaling mediators [31-33]. Exposure of astrocytes to PDGF-BB resulted in a time-dependent increase of p65 subunit of NFκB in the nucleus with a concomitant transient increase in cytosolic pIκB (Figure 6A). Validation of the role of NFκB was further determined by pretreating astrocytes with the IκB pathway inhibitor (SC514), followed by PDGF-BB treatment for 24 h. As shown in Figure 6B, SC514 inhibited PDGF-BB-mediated induction of MCP-1, thereby underscoring the role of NFκB in this process.

To corroborate the findings found using the pharmacological inhibitors these cells were then transduced with either WT or DN adenoviral constructs of NFκB. As shown in Figure 6C, transduction with DN form of NFκB resulted in inhibition of PDGF-BB-mediated induction of MCP-1. Transduction with the WT NFκB construct, however, and as expected, demonstrated PDGF-BB-mediated induction of MCP-1. Together, these findings underpin the role of NFκB in PDGF-BB-mediated induction of MCP-1 in astrocytes.

Since NFκB is a transcription factor that mitigates its effects on target genes by binding to promoter regions, we next sought to confirm the binding of NFκB with MCP-1 promoter in its natural chromatin context by ChIP assay to reveal active sites accessible to NFκB. A172 cells were treated with PDGF-BB for 1 h followed by RNA extraction and processed using a ChIP analysis kit. These experiments revealed increased binding of NFκB to the MCP-1 promoter in A172 cells treated with PDGF-BB (Figure 6D,E) and, along with preceding data, substantiate the role of NFκB in PDGF-BB-mediated regulation of MCP-1 in astrocytes.

### MAPK and PI3K/Akt cell signaling pathways lie upstream of PDGF-BB induced NFκB in astrocytes

**Figure 7:**
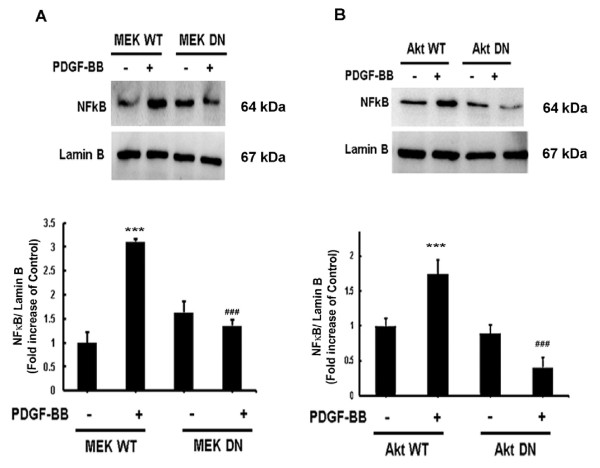
**Mitogen-activated protein kinase (MAPK) and phosphatidylinositol 3-kinase (PI3K)/Akt cell signaling pathways lie upstream of platelet-derived growth factor (PDGF)-BB induced nuclear factor κB (NFκB) in astrocytes. **The nuclear fractions of A172 cells transfected with wild-type (WT) or dominant-negative (DN) forms of mitogen-activated protein (MAP) kinase kinase (MEK) and Akt were subjected to western blot analysis for NFκB. (**A**) Transfection with DN-MEK but not WT-MEK resulted in abrogation of PDGF-BB-mediated induction of NFκB phosphorylation. (**B**) Transduction with DN-Akt but not WT-Akt also resulted in abrogation of PDGF-BB-mediated induction of NFκB phosphorylation. All the data are presented as mean ± SD of three individual experiments. ***P <0.001 versus control group, ###*P *<0.001 versus PDGF-BB-treated group.

Having determined the involvement of ERK1/2, JNK and p38 MAPKs and NFκB in PDGF-BB-mediated MCP-1 the next logical step was to examine whether there existed a link that could tie together the activation of MAP kinase and Akt pathways with NFκB. Astrocytes were transfected with either the WT or DN constructs of MEK prior to PDGF-BB treatment as described before. Nuclear fractions were extracted and NFκB phosphorylation was assessed via western blotting. PDGF-BB-mediated induction of NFκB was attenuated by DN-MEK, but not by WT-MEK construct (Figure 7A). To confirm the role of PI3K/Akt in PDGF-BB-mediated NFκB, A172 cells were transduced with adenoviral constructs containing either WT or DN forms of Akt, treated with PDGF-BB and assessed for NFκB expression. As shown in Figure 7B, cells transduced with DN Akt construct failed to upregulate NFκB unlike the cells transduced with the WT Akt construct. These findings thus linked PDGF-BB-mediated activation of MAP kinase and Akt pathways to the downstream activation of NFκB.

### MCP-1 released from astrocytes increases monocyte transmigration in human brain microvascular endothelial cells

**Figure 8:**
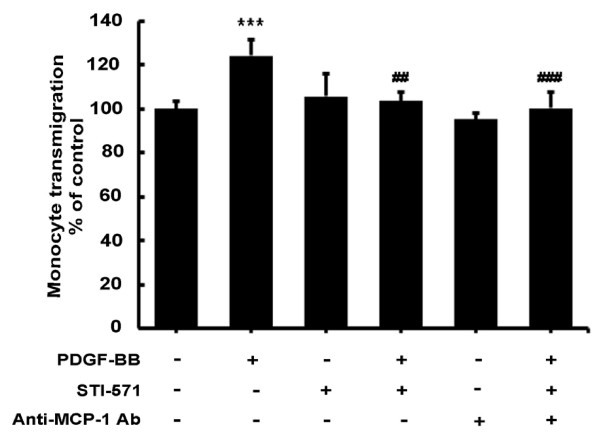
**Platelet-derived growth factor (PDGF)-BB-induced monocyte transmigration and permeability is mediated by monocyte chemoattractant protein 1 (MCP-1). **Human brain microvascular endothelial cells (HBMECs) were exposed for 24 h to supernatants from A172 cells treated with PDGF-BB with or without STI-571. MCP-1 neutralizing antibody was added to PDGF-BB-treated media prior to HBMEC exposure for 24 h. Human monocytes were added to the top transwell for 24 h followed by assessment of transmigration. Exposure of HBMECs to supernatants from A172 cells treated with PDGF-BB resulted in increased permeability and enhanced monocyte transmigration, which was abrogated by MCP-1 neutralizing antibody. All the data are presented as mean ± SD of three individual experiments. ***P <0.001 versus control group; ##*P *<0.01, ###*P *<0.001 versus PDGF-BB-treated group.

Having determined the induction of MCP-1 by PDGF-BB, the next step was to explore the functional relevance of this upregulation. Since MCP-1 is a known chemoattractant, we hypothesized that PDGF-BB-induced MCP-1 released from the astrocytes could, in fact, act on neighboring endothelial cells altering their function. Human astrocytes were exposed to PDGF-BB for 2 h then replaced with fresh media and incubated for 24 h. Endothelial cells were grown on the upper compartment of transwell plates and spent media was added to the lower compartment overnight. Labeled human monocytes were added to the upper compartment for 24 h and monocyte transmigration was assessed. As shown in Figure 8, conditioned media from primary astrocytes cells treated with PDGF-BB resulted in a significant increase in monocyte transmigration of endothelial cells, an effect that was blocked by PDGF-R blocker STI-571.

Since monocytes express the MCP-1 receptor CCR2 [34,35], the next step was to determine whether increased MCP-1 was also able to enhance monocyte migration. Also shown in Figure 8A, conditioned media from primary astrocytes cells treated with PDGF-BB resulted in a dramatic increase in monocyte transmigration of endothelial cells and this effect was ameliorated in conditioned media treated with the MCP-1 neutralizing antibody. These findings thus confirm that MCP-1 was involved in PDGF-BB-mediated disruption of the endothelial barrier permeability and not only underpin the role of MCP-1 in BBB breakdown, but reveal a vital role that activated astrocytes play in BBB disruption and HAND pathogenesis.

## Discussion

Antiretroviral therapies have proven highly effective in controlling systemic viral infection, thus leading to increased longevity in patients with AIDS. The inability of some of these drugs to cross the BBB results in slow and smoldering infection in the CNS. Subsequently, the brain becomes a sanctuary of virus-induced toxicity leading to increased prevalence of HAND in HIV-infected individuals. One of the hallmark features of HAND is increased astrogliosis comprising of increased numbers of activated astrocytes, culminating ultimately into increased neuronal degeneration. It is well recognized that activation of astrocytes leads to the release of a barrage of inflammatory mediators such as PDGF-BB. PDGF-BB has been implicated in a variety of pathological conditions; however, its role in HIV pathogenesis remains less clear.

In the present study we demonstrate that HIV-1-mediated induction of MCP-1, a potent chemokine vital to the sustained proinflammatory response in HIV-1 pathogenesis, is regulated by PDGF-BB. To determine the implication of increased PDGF-BB we demonstrated that PDGF-BB treatment of human astrocyte cell line and primary cultures resulted in induction of MCP-1 and this process was mediated via the binding of PDGF-BB to its cognate PDGF-Rβ. These findings are consistent with the reports on PDGF-mediated induction of MCP-1 in other cell types such as the fibroblast cell line and smooth muscle cells [36,37]. In our efforts to dissect the upstream signaling events that are involved in MCP-1 release from astrocytes, using both the pharmacological as well as genetic approaches we demonstrated the role of MAPK and PI3K/Akt in PDGF-BB-mediated induction of MCP-1 from astrocytes. The involvement of MAPK and PI3K/Akt pathways in the induction of MCP-1 expression are in agreement with the role these pathways play in induction of this chemokine in other cell types including osteoblasts, mesangial cells and endothelial cells [38-40]. The transcription factor, NFκB is known to play a key role in PDGF-BB signaling and also in the expression of proinflammatory cytokines/chemokines including MCP-1 [31,33,41]. Consistent with other reports, our studies also revealed that PDGF-BB-mediated induction of MCP-1 involved both NFκB activation and its binding to the MCP-1 promoter.

It was next of interest to explore the functional relevance of PDGF-BB-mediated induction of MCP-1. Based on the proximity of astrocytes to the endothelial barrier, we rationalized that induced expression of MCP-1 in PDGF-BB treated astrocytes could play a role in barrier integrity. Intriguingly, conditioned media from PDGF-BB treated astrocytes did indeed increase monocyte transmigration and this effect was attributable to MCP-1 as demonstrated in the blocking antibody experiments. This role of MCP-1 is in agreement with the findings reported by Eugenin *et al.* who have demonstrated that HIV-infected leukocyte transmigration across a tissue culture model of human BBB involved MCP-1 [42]. In addition to disrupting the barrier permeability, MCP-1 also manifested increased monocyte recruitment. This latter function is in keeping with its known roles both as a chemoattractant and as a biomarker of HIV neuropathogenesis [42,43].

The function of MCP-1 demonstrated in this study can have ramifications in the pathogenesis of HAND. Based on the proximity of astrocytes to the endothelium and their ability to secrete both PDGF-BB and the chemokine MCP-1 as well as their abundance in the CNS, it can be argued that during HIV-1 infection, viral proteins can initiate a toxic cascade that can be self-perpetuating. HIV-1/HIV-1 Tat can trigger increased expression of PDGF-BB, which in turn, can lead to increased MCP-1 expression that can manifest as an amplified influx of monocytes into the CNS. PDGF-BB has already been shown to disrupt the BBB [19] and a similar function has been demonstrated for MCP-1 [42]. Therefore the release of both these mediators can independently disrupt the endothelial barrier while enhancing neuroinflammation [19,42], which can have serious ramifications in HAND.

In summary, our studies have mapped out a detailed molecular pathway of PDGF-BB-mediated MCP-1 expression in astrocytes involving ERK1/2, JNK MAPK activation, with the subsequent activation of NFκB resulting in increased MCP-1 expression, ultimately leading to increased monocyte transmigration and increased permeability in the brains of individuals infected with HIV.

## Competing interests

The authors declare that they have no competing interests.

## Authors’ contributions

CB-B designed research, performed research and wrote the manuscript; HY designed the research and wrote the manuscript; GH performed research; SB planned and designed the research and wrote the manuscript. All authors have read and approved the final version of the manuscript.
